# Role of the JAK/STAT Pathway in Cervical Cancer: Its Relationship with HPV E6/E7 Oncoproteins

**DOI:** 10.3390/cells9102297

**Published:** 2020-10-15

**Authors:** Adriana Gutiérrez-Hoya, Isabel Soto-Cruz

**Affiliations:** 1 Molecular Oncology Laboratory, Cell Differentiation and Cancer Research Unit, FES Zaragoza, National University of Mexico, Batalla 5 de Mayo s/n Col. Ejército de Oriente, Mexico City 09230, Mexico; agutierrezho@conacyt.mx; 2 Cátedra CONACYT, CONACYT, Avenida Insurgentes Sur 1582, Col. Crédito Constructor Del. Benito Juárez, Mexico City 03940, Mexico

**Keywords:** signal transducer and activator of transcription, STAT inhibitors, signaling pathway, cervical cancer, HPV

## Abstract

The janus kinase (JAK)/signal transducer and activator of transcription (STAT) signaling pathway is associated with the regulation of essential cellular mechanisms, such as proliferation, invasion, survival, inflammation, and immunity. Aberrant JAK/STAT signaling contributes to cancer progression and metastatic development. STAT proteins play an essential role in the development of cervical cancer, and the inhibition of the JAK/STAT pathway may be essential for enhancing tumor cell death. Persistent activation of different STATs is present in a variety of cancers, including cervical cancer, and their overactivation may be associated with a poor prognosis and poor overall survival. The oncoproteins E6 and E7 play a critical role in the progression of cervical cancer and may mediate the activation of the JAK/STAT pathway. Inhibition of STAT proteins appears to show promise for establishing new targets in cancer treatment. The present review summarizes the knowledge about the participation of the different components of the JAK/STAT pathway and the participation of the human papillomavirus (HPV) associated with the process of cellular malignancy.

## 1. Introduction

The Janus kinase (JAK)/signal transducer and activator of transcription (STAT) pathway plays a central role in immune responses, cell proliferation, differentiation, and survival. Overexpression and overactivation of the components of the JAK/STAT pathway are associated with the development of different types of cancer. One of them, cervical cancer [1], is the cancer with the fourth highest incidence and mortality worldwide (Globocan 2018); in 2018, Globocan reported 569,847 cases of cervical cancer and 311,365 related deaths. Cervical cancer remains a public health problem affecting middle-aged women, especially in developing countries [2,3]. Persistent high-risk human papillomavirus (HPV) infection is the main trigger of the development of cervical cancer; the most common high-risk HPV types are 16, 18, 31, 33, 35, 45, 52, and 58. Of these, two types (HPV16 and HPV18) cause between 70% and 72% of invasive cervical cancers [4]. High-risk papillomaviruses have two oncoproteins, i.e., E6 and E7, necessary for the establishment and progression of cervical cancer.

Effective prophylactic vaccines against common oncogenic HPV types 16 and 18 are available. There are currently three types of licensed prophylactic HPV vaccines: Gardasil^®^ (quadrivalent vaccine targeting HPV6, HPV11, HPV16, and HPV18), Cervarix^®^ (bivalent vaccine targeting HPV16 and HPV 18), and Gardasil 9^®^ (vaccine targeting HPV6, HPV11, HPV16, HPV18, HPV31, HPV33, HPV45, HPV52, and HPV58), approved in 2006, 2007, and 2014, respectively. The introduction of prophylactic HPV vaccines has shown a reduction in the prevalence of HPV and HPV-related diseases, such as genital warts, cervical intraepithelial neoplasia, and cervical cancer [5].

A meta-analysis of the impact of vaccination against HPV that includes data from 60 million people with up to eight years of post-vaccination follow-up shows that the prevalence of HPV16 and 18 decreased significantly to 83% and 66% in girls from 13 to 19 years old and women from 20 to 24 years old, respectively. Moreover, cervical intraepithelial neoplasia grade 2 decreased by up to 51% and 31% in girls aged 15–19 years and women aged 20–24 years, respectively. Vaccination also led to a decrease in the diagnosis of anogenital warts in girls, boys, women, and men. However, although vaccines are effective in protecting against HPV infection, they provide limited benefits in eliminating pre-existing infections, and HPV vaccines do not protect against all types of HPV that can cause cancer [6]. It is also important to note that, worldwide, the average age of cervical cancer diagnosis is 53 years (with a range of 44–68 years) and the global average age of death from cervical cancer is 59 years (with a range of 45–76 years). Thus, new prevention and treatment strategies must be designed [2]. There are many reports about epidemiology and molecular changes during HPV infection, as well as about the molecular biology of E6/E7 oncogenes. Currently, research is underway to understand the molecular signaling pathways in cervical cancer. The best-known targets of E6 and E7 are pRb and p53, respectively, proteins with regulatory functions. However, they are not their only targets; E6 and E7 can also regulate epigenetic marks, splice changes, generate regulatory RNAs—regulators of the transcription among other changes that allow the virus to proliferate in an uncontrolled manner—and generate cell transformation and carcinogenesis [7,8,9,10]. In this context, multiple publications show how HPV infection increases the activity of proto-oncogenic transcription factors such as those involved in the JAK/STAT pathway. This pathway allows direct communication from transmembrane receptors to the nucleus and is used in normal cells of the immune system to respond to a wide variety of cytokines, hormones, and colony-stimulating factors. Moreover, JAK/STAT signaling has been found, constitutively active in several solid tumors, to contribute to cancer progression and metastatic development [11]. In this review, we focus on the antecedents involving the JAK/STAT pathway and its relationship with E6/E7 HPV oncoproteins in cervical cancer progression.

## 2. JAK/STAT Pathway

**Figure 1:**
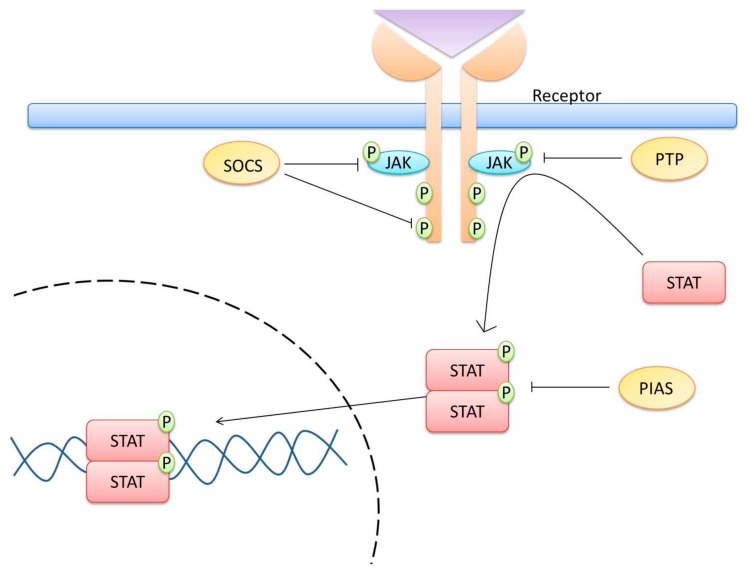
The Janus kinase (JAK)/signal transducer and activator of transcription (STAT) signaling pathway. Membrane cytokine receptors have cytoplasmic tails in which inactive JAKs associate constitutively. The interaction of cytokines or growth factors with their receptors (type I and II) induces dimerization/oligomerization of these receptors. It should be mentioned that some cytokine receptors, such as GHR and EpoR, show pre-formed dimers. In both cases, the interaction between the cytokine and its receptor induces a conformational change in the cytoplasmic domain. This interaction results in the juxtaposition of JAKs, leading to their autophosphorylation or transphosphorylation by other JAKs or other families of tyrosine kinases. The activated JAKs then phosphorylate the receptor’s cytoplasmic tails on tyrosine residues, creating sites that allow the binding of other signaling molecules that contain an SH2 domain (such as STAT proteins). Cytoplasmic STATs then bind to phosphorylated receptors, becoming substrates for JAKs, which phosphorylate STATs on highly conserved tyrosine residues. After their phosphorylation, STATs form homodimers or heterodimers that are capable of translocating to the nucleus and activating gene transcription. The JAK/STAT pathway is negatively regulated by the suppressors of cytokine signaling (SOCS), as well as by the protein inhibitor of activated STAT (PIAS) and protein tyrosine phosphatases (PTPs).

The JAK/STAT pathway is an essential signaling pathway involved in progenitor cell maintenance, hematopoiesis, immune regulation, and recently tumor processes [1,11,12]. This pathway participates in physiological functions such as cell proliferation, differentiation, angiogenesis, and apoptosis [1,13]. The pathway mediates signals from a wide variety of cytokines and growth factors. An important role of this pathway is the transfer of signals from cell membrane receptors to the nucleus. Type I and II cytokine receptors constitutively associate with JAKs, and the interaction with the respective cytokines induces dimerization/oligomerization of the receptor that results in the juxtaposition of JAKs; through homodimeric or heterodimeric interactions, the JAKs are phosphorylated either by autophosphorylation or transphosphorylation by other JAKs or other families of tyrosine kinases. The activated JAKs then phosphorylate the cytoplasmic tyrosine residues of the receptor, creating binding sites that allow the binding of other signaling molecules that contain a Src homology 2 (SH2) domain, such as STATs, and then STATs are phosphorylated by JAKs. Phosphorylated STATs form either homodimers or heterodimers that are capable of translocating to the nucleus and acting as transcription factors (Figure 1). However, there is evidence that some cytokine receptors, such as EpoR, GHR, gp130, LEPR, IL-2Rβ, IL-9Rα, and γc, are already pre-formed dimers, and their binding to cytokines induces conformational changes that transmit an activation signal to the associated JAKs [14,15,16,17,18].

STAT dimers bind to specific promoter sequences and modulate gene transcription [19,20,21,22]. However, STATs can also be phosphorylated by G protein-coupled receptors (GPCRs), such as the receptor for CCR5 (MIP-1, RANTES receptor) by agonist-dependent activation of the angiotensin II receptor type 1 (AGTR1), and by non-receptor tyrosine kinases such as the Src family of kinases; BCR-Abl and TEL-Abl oncoproteins; or viral oncoproteins such as v-Src, v-Abl, v-Sis, and v-Eyk [13,22,23,24,25].

## 3. Central Players for the Activation and Regulation of the JAK/STAT Pathway

The basic components of the JAK/STAT pathway are ligands (cytokines or growth factors), cytokine receptors (types I and II), JAKs, STATs, and suppressors of cytokine signaling (SOCSs) [19].

All type I and II cytokine receptors selectively associate with JAK (JAK1, JAK2, JAK3, or TYK2) to transmit their signals to the cytoplasm because they lack intrinsic tyrosine kinase activity (Table 1). The interaction of the cytokine receptor is critical for the transphosphorylation of JAK and the subsequent recruitment of one or more STATs to be phosphorylated [26,27].

Janus kinases. In humans, there are four members of the JAK family: JAK1, JAK2, JAK3, and TYK2. Each member of JAK comprises several distinct domains: an N-terminal FERM (protein 4.1, ezrin, radixin, and moesin) domain that mediates interaction with receptor complexes; an SH2 domain (Src 2 homology) that binds to phosphotyrosine residues; a pseudokinase domain (domain with homology to the protein tyrosine kinase domain, but without catalytic function) that has a regulatory role; and a protein tyrosine kinase (PTK) domain located at the C-terminus (responsible for the phosphorylation of particular substrates downstream) [19,20,21].

Signal transducer and activator of transcription. The STAT family has seven STAT proteins: STAT1 (STAT1α and STAT1β are two proteins encoded by the same gene, but the result of alternative splicing), STAT2, STAT3, STAT4, STAT5a, STAT5b, and STAT6. These proteins control biological functions such as cell differentiation, proliferation, inflammation, and apoptosis [28,29,30]. After being phosphorylated, STAT proteins form a homo/heterodimer before translocating to the nucleus and act as a transcription factor [31]. Each set of homo- or heterodimers have different target genes, and that is the reason they can control different processes.

Cytokine signaling suppressors. Activation of the JAK/STAT pathway can be controlled by suppressors of cytokine signaling (SOCS). The SOCS protein family consists of eight members: SOCS1, SOCS2, SOCS3, SOCS4, SOCS5, SOCS6, SOCS7, and the protein-inducible SH2 domain cytokines (CIS or CISH). Each SOCS protein has a central SH2 domain, an amino-terminal domain, and a 40 amino acid carboxy-terminal module known as the SOCS box, and SOCS1 and SOCS3 possess a kinase inhibitory region (KIR). The SH2 domain binds directly to the phosphorylated tyrosine of activated JAKs and blocks STAT recruitment and activation. The inhibitory region of the protein blocks the function of JAK because it serves as a pseudosubstrate. The SOCS box interacts with several enzymes of the ubiquitination machinery for the ubiquitination and degradation of JAKs through the proteasome [19,32].

Other regulators of the JAK/STAT pathway are the protein inhibitor of activated STAT (PIAS) and protein tyrosine phosphatases (PTPs). The PIAS family consists of five members: PIAS1, PIASxα, PIASxβ, PIAS3, and PIASy, which can regulate transcription in four ways: (1) by blocking the DNA-binding activity of a transcription factor; (2) by recruiting other co-regulators (such as histone deacetylases); (3) by promoting the SUMOylation of a transcription factor; and (4) by sequestering transcription factors. There are also different PTPs: CD45, SHP1, SHP2 PTP1B, T cell PTP (TC-PTP), PTPRT, and PTPBL, which function by dephosphorylating the tyrosine residues involved in the activation of the JAK/STAT signaling pathway to inhibit it. However, it has been observed that SHP2 can positively regulate JAK/STAT signaling. Studies suggest that SHP2 could dephosphorylate JAK2 at residue Y1007, preventing the association of JAK2–SOCS1, and thus avoiding the degradation of JAK2 to promote the activation of STAT5, as well as preventing the dephosphorylation of JAK2 at Y570 and promoting the activation of STAT3 [19,33,34,35,36,37,38]. Mutations in the *PTPN11* gene that codes for the SHP2 protein have been observed in Noonan syndrome; these mutations cause an overactivation of SHP2 and are associated with hyperactivation of the extracellular-signal-regulated kinase (ERK1/2) pathway. It has recently been found that SHP2 can play a dual role in the different signaling pathways associated with the development of cancer; for example, SHP2 has a negative regulatory effect on the JAK/STAT3 signaling pathway. However, different studies have suggested that the phosphorylation of Y759 of gp130 is a binding site for SHP2, which promotes signaling through the Grb2 and Gab proteins, which function as adapter proteins that induce RAS/ERK activation. RAS/MAPK and PI3K/Akt are signaling pathways involved in survival, proliferation, malignant transformation, and drug resistance [38,39,40,41]. Therefore, SHP2 could be considered a potential molecular target for cancer treatment. SHP2 is associated with different diseases, and its upregulation has been observed in various cancers (e.g., leukemia, lung and breast cancers, and neuroblastomas), which is the reason SHP2 inhibitors are investigated as a strategy for cancer therapy [38].

## 4. The JAK/STAT Pathway Is Involved in T Helper Cell Differentiation

Some members of the JAK/STAT pathway have been widely explored in the context of cancer. Several members of the STAT family have been linked to tumor initiation and progression, while others participate in the antitumor defense and maintenance of an effective and long-term immune response [21]. A key feature in the interaction of malignant cells with the tumor microenvironment is their ability to evade or even suppress antitumor immune responses. It is well-documented that the differentiation of naïve T cells in various subpopulations depends mainly on the action of cytokines and the signaling pathways that turn on; in this context, the JAK/STAT pathway plays an essential role in the differentiation of CD4 T cells and the action of these on the immunological process. Hence, T cells provide a unique opportunity to understand how the JAK/STAT pathway is used in healthy cells to achieve proliferation and survival in comparison with that observed in cancer cells.

Helper T (Th) cells can differentiate into multiple effector subpopulations, including Th1, Th2, Th17, and regulatory T cells, and these subpopulations are very significant in host health and disease. For example, Th1 cells are characterized by their production of IFNγ and are important for the protective immune response of intracellular bacteria and viruses. Th2 cells are characterized by the production of IL-4, IL-5, and IL-13, and are necessary for the protection of extracellular parasites. Th17 cells secrete a distinctive set of immunoregulatory cytokines, including IL-17A, IL-17F, IL-22, and IL-21, which are important in extracellular and fungal protection. Finally, Treg cells are characterized by the production of TGF-β and IL-10, which are necessary for the maintenance of immune tolerance and to regulate the activation of the immune system [42,43].

**Figure 2:**
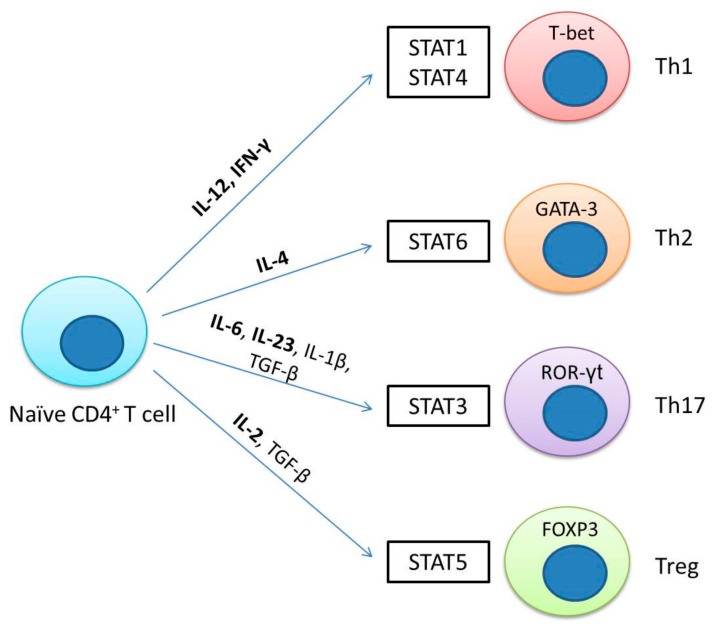
JAK/STAT signaling drives the differentiation of T helper cells (Tregs). T cell differentiation from naïve cells into the various functional subsets depends on the presence of specific cytokines and the activation of a particular STAT protein. Cytokine receptors can activate several other STATs, which may depend on the cell type and the concentration of the cytokine.

The differentiation of T helper (Th) cells into multiple effector subpopulations requires the recognition of a major class II histocompatibility complex loaded with an antigen, interaction with costimulatory molecules, and cytokine signaling. Cytokines play a key role in the induction of signaling and transcription networks, and the JAK/STAT pathway is necessary for the differentiation of Th cells (Tregs) [27,44,45]. For example, Th1 polarization is driven by IL-12 and IFN-γ through the activation of JAK2/TYK2 for STAT4 and JAK1/JAK2 for STAT1. For the Th2 phenotype, IL-4 signals through JAK1/3 to activate STAT6. For the Th17 subpopulation, the cytokines TGF-β and IL-6 are required, and IL-6 signals through JAK1/2 for the activation of STAT3. Finally, for the induction of Tregs, the cytokines TGF-β and IL-2 are necessary, of which IL-2 signals through the JAK1/3 path to activate STAT5 (Figure 2). Additionally, cytokines can activate other STATs, which may depend on the cell type and the concentration of the cytokine. For example, IL-12 can activate STAT3 and STAT5, IL-6 can activate STAT1 and STAT5, while IL-2 can activate STAT3 and STAT1 [46,47,48].

It is important to mention that, for the polarization of T cells and each lineage, two or more types of transcription factors are required. Essential transcription factors in the polarization of Th cells are as follows: T-bet for Th1 cells, GATA-3 for Th2, RORγT for Th17, and FOXP3 for iTreg [49,50,51,52,53,54,55,56,57,58].

The JAK/STAT pathway is involved in the proliferation and survival of the cells of the immune system. However, the overactivation of JAK/STAT proteins, as well as the reduction of the different SOCS, are associated with proliferation, progression, metastasis, and survival in various types of tumor cells. The JAK/STAT pathway is also associated with the development of tumor tolerance, due to a strong correlation between JAK/STAT hyperactivation and an increase in the genes involved in the regulatory function of Tregs [21,59].

## 5. JAK/STAT Pathway and Cervical Cancer

Recently, the implication of the activation or inhibition of the JAK/STAT pathway in the development of cancer has become important; some types of cancer, such as breast, liver, lung, and pancreas, have received more attention, and thus there is more basic and clinical research regarding them [60]. However, the role of the JAK/STAT pathway in cervical cancer remains poorly understood; thus, it is essential to understand the role of each of the components of the JAK/STAT pathway in the development and control of this neoplasia, which represents a public health problem in developing countries.

### 5.1. Role of STAT Proteins in Cervical Cancer

More than 90% of cervical cancer samples contain high-risk HPV DNA, and reports exist that the E5, E6, and E7 oncoproteins can alter multiple signaling pathways, such as phosphoinositide 3-kinase (PI3K)/protein kinase B (Akt), Wnt, and Notch, essential pathways in the initiation and maintenance of HPV-associated cancers. However, it has recently been found that HPV may also have an impact on different components of the JAK/STAT pathway and its inhibitors [61,62,63].

#### 5.1.1. STAT1

STAT1 is activated in response to different cytokines, mainly interferons (IFNs). The signal through IFNs activates STAT2 and STAT1; subsequently, the STATs form heterodimers pSTAT1/pSTAT2 or homodimer pSTAT1, which move to the nucleus and bind to gamma interferon activation site (GAS) elements. However, the effect of STAT1 is not always linked to STAT2. For example, in colorectal carcinoma and squamous cell carcinoma of the esophagus, STAT1 is associated with a good prognosis, which is not the case for STAT2 [64,65], probably because STAT1 has multiple effects on cell cycle inhibition by the regulation of p21, p27, and the cyclin D1/Cdk4 complex [66]. STAT1 plays a central role in the sensitization to apoptosis, thanks to its regulation of the expression of pro-apoptotic proteins and the cell death receptor, as well as its ligands, which mediate the activation of caspases [67,68,69]. Furthermore, STAT1 is essential for the activation of the immune system through the recognition of the major histocompatibility complex (MHC-I), as STAT1 is vital for the positive regulation of this complex, which is necessary for the recognition of cytotoxic T cells [70]. On the other hand, STAT1 can inhibit angiogenesis by inhibiting hypoxia inducible factor alpha (HIF-1α) and vascular endothelial growth factor (VEGF-A), thereby promoting a decrease in tumor growth [71,72]. However, recently, STAT1 has been shown to be associated with poor survival in some cancers [73,74] and has been reported to be responsible for the immunosuppressive tumor microenvironment [75], resistance to therapy, and the potential for metastasis [76,77], and the non-phosphorylated form STAT1 promotes apoptotic resistance by repressing the expression of *FAS* and *BAD* genes [78]. These data could implicate non-phosphorylated STAT1 and the probable participation of STAT3, as well as the formation of STAT1/STAT3 heterodimers in apoptosis resistance of tumor cells. It is known that type I interferons can also activate STAT3. The formation of STAT1/STAT3 heterodimers does not suppress STAT1 tyrosine phosphorylation or its translocation to the nucleus, but instead suppresses the formation of STAT1 homodimers that bind to DNA. Thus, IFN-activated STAT3 inhibits STAT1, downregulating the induction of inflammatory mediators [79].

Regarding the role of STAT1 in cervical cancer, its expression in cervical lesions and cervical cancer has been elucidated. Rajkumar’s group found STAT1 overexpression in cervical intraepithelial neoplasia (CIN) 1/2, a decrease in CIN3/cervical carcinoma in situ (CIS), and a significant increase in invasive cancers [80]. Consistent with these results, Ding’s group found significantly higher levels of STAT1 in cervical cancer samples compared with non-tumor tissues [81]. Additionally, Yi’s group found that STAT1 is upregulated in CIN1, CIN2, CIN3, and cervical cancer, but the expression of STAT1 has no significant effect on overall survival [82].

Furthermore, there are reports regarding the effect of HPV on STAT1. For example, keratinocytes infected with the E6/E7 oncoproteins of high-risk HPV, individually or together, show a decrease in the expression of STAT1α/β [83,84]. HPV16 E6 is capable of reducing the amount of STAT1, as well as binding to IFN-stimulated response elements (ISREs). Meanwhile, E6 and E7 decrease the translocation of STAT1 to the nucleus [85]. According to Hong’s group, a decrease in STAT1 is necessary for the amplification of the viral genome in the early stage of infection, perhaps owing to its ability to suppress interferon-inducible genes, thus evading the immune system [84]. Other reports support the importance of STAT1 in the control of tumor growth; for example, Lei et al. showed that the treatment of HeLa cells with cisplatin increases the expression of STAT1 and that the silencing of STAT1 with small interfering RNA (siRNA) promotes cell proliferation and rescues the tumor cells treated with cisplatin, which shows that the expression of STAT1 is essential for the induction of death in tumor cells [86]. Consistent with this report, Buttarelli et al. demonstrated that patients with locally advanced cervical cancer sensitive to chemoradiation treatment have higher levels of STAT1 compared with resistant cases, which suggests that STAT1 may contribute to improved radiosensitivity [87].

These findings indicate that STAT1 could have a dual role in HPV infection and tumorigenesis, playing a protective role in the early phase of HPV infection, but functioning as a proto-oncogene in the invasive stages. However, more studies are needed to detail the role and involvement of STAT1 in infection and transformation processes.

#### 5.1.2. STAT2

STAT2 is a necessary transcription factor in the signaling pathway for type I interferons (IFNα and IFNβ). After the interaction of type I IFNs with their receptor, tyrosine kinases JAK1 and TYK2 are activated to phosphorylate the proteins STAT1 and 2. Phosphorylated STAT1 and STAT2 form heterodimers and bind to IRF9, in order to transcribe the genes induced by IFN (ISG) [88]. STAT2 is necessary for the antiviral, immunomodulatory, antiapoptotic, and antiproliferative effects of IFN-I. Yue et al. showed that STAT2-deficient mice have increased tumor growth due to a lack of response to type I interferons [89]. However, Ho et al. found that STAT2 can function as an inhibitor of STAT1. They showed that unphosphorylated STAT2 can bind activated STAT1 to specifically inhibit the nuclear translocation of STAT1 in response to IFN-γ [90]. The role of STAT2 in cancer is controversial; some reports implicate STAT2 in the carcinogenesis process, proposing that STAT2 can increase IL-6 secretion, and that this in turn activates STAT3, thus promoting tumorigenesis [88].

In cervical cancer, Liang et al. found that the biopsies with CIN had a higher expression of STAT2 than the cervicitis biopsies, but they did not observe that the increase in STAT2 was proportional to the severity of cervical lesions. Therefore, they suggested that the increased expression of STAT2 begins in premalignant dysplasia and remains in cervical cancer. They also found no difference in the five-year survival between cervical cancer patients with low and high STAT2 expression [91]. On the other hand, it is known that HPV can indirectly inhibit STAT2; the E6 oncoprotein can interact directly with Tyk2 and prevent its association with IFNAR1, thus preventing the phosphorylation of STAT1 and STAT2 [92]. The E7 oncoprotein of HPV16 binds to IFR9, thereby inhibiting its binding to STAT1/STAT2 heterodimers and preventing the formation of the ISGF3 complex and its translocation to the nucleus [93]. It seems that HPV uses this strategy to block the action of IFNs. However, the role of STAT2 in cervical cancer remains poorly understood and further analysis is needed to determine whether its activation in cervical cancer cells could be a good or a bad prognostic factor.

#### 5.1.3. STAT3

STAT3 is one of the most studied members of the family. This protein is activated through receptor tyrosine kinases (RTKs) such as the epidermal growth factor receptor (EGFR) and various cytokine receptors such as IL-6R, IL-11R, IL-23R, and IL-10R. Canonical activation of STAT3 requires phosphorylation of its tyrosine 705 by JAK1, and phosphorylation of STAT3 results in its dimerization and nuclear translocation. However, STAT3 is also activated by phosphorylation of serine 727 for its translocation to mitochondria, and this phosphorylation is usually regulated by protein kinase C, mitogen-activated protein kinases, and CDK5. In addition, STAT3 has a third activation mechanism, namely, is its acetylation in lysine 685, which improves its stability and transcriptional activity [28,94,95].

**Figure 3:**
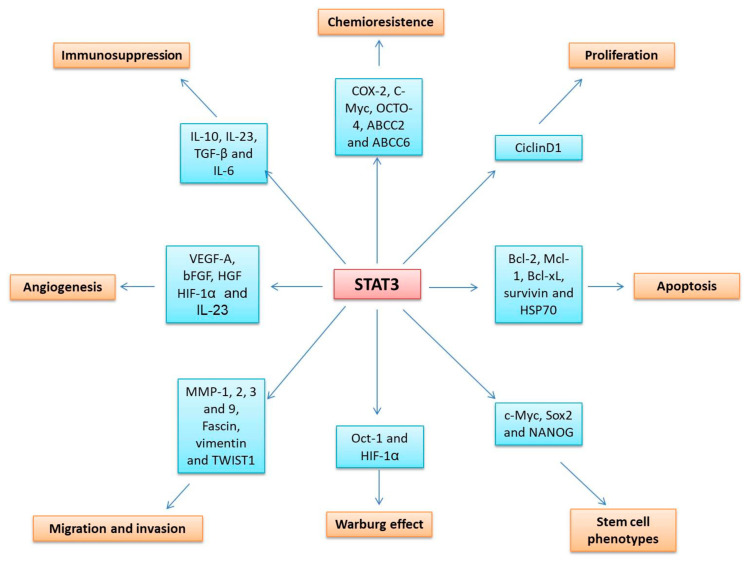
STAT3 regulates the transcription of gene sets involved in cancer development.

STAT3 hyperactivation has been reported in several types of solid tumors, for example, cancer of the cervix, bladder, bone, breast, brain, colon, esophagus, head and neck, kidney, liver, lung, ovary, pancreas, prostate, stomach, and uterus, as well as hematological cancers such as acute myeloid leukemia and multiple myeloma. Common causes of STAT3 overactivation are the presence of large amounts of cytokines and growth factors such as IL-6, IL-10, EGFR, and platelet-derived growth factor receptors (PDGFRs); the absence of negative regulatory molecules such as SOCS1 and SOCS3 and the activation of non-receptor protein tyrosine kinases such as src and BCR-Abl [13]. STAT3 plays a crucial role in tumor development, thanks to its ability to regulate the transcription of the genes involved in the cell cycle (*CCND1* (cyclin D), *CCNE1* (cyclin E), and *RAC1*), cell survival (*BCL2*, *BCLXL*, *HSP70* and *FAS*), metabolism (*OCT1*, *HIF1A*), chemoresistance (*COX-2*, *MYC*, *OCTO-4*, *ABCC2*, and *ABCC6*), immunosuppression (*IL-10*, *IL-23*, *IL-6*, and *TGFB*), angiogenesis (*VEGFA* and *bFGF*), migration and invasion (*MMP1*, *2*, *3* and *TWIST1*), and stem cell phenotypes (*MYC*, *SOX-2*, and *NANOG*), among other mechanisms (Figure 3) [96,97].

STAT3 in cervical cancer has acquired importance from observations showing that its presence and activity are associated with the malignancy of cervical lesions [98]. High-risk HPV-positive cells show a higher amount of active STAT3 (pY705) compared with HPV-negative cells [99]. The level of active STAT3 is also associated with the number of copies of the HPV genome. Additionally, HPV-positive cervical tumor cells are capable of producing large amounts of IL-6 for autocrine signaling and for increasing STAT3 activation [100]. It is well-documented that STAT3 is an essential regulator in cell transformation and that different viruses have strategies to stimulate its signaling and activation [101].

We now know that the expression of the HPV genome depends mainly on host transcription factors, and some transcription factors such as AP-1, NF-κB, and also STAT3 may play a regulatory role in HPV infection owing to the presence of its cis-related elements in the upstream regulatory regions (URRs) and its association with the degree of carcinogenesis [102]. Arany et al. suggested that STAT3 could bind to HPV16 upstream of the URR, driving the expression of E7 [103]. However, STAT3 also affects the expression of E6; different studies show the inhibition of STAT3 with the STAT3 inhibitor cryptotanshinone or STAT3-specific siRNA or its blocking of tyrosine phosphorylation by AG490 or curcumin as a consequence the reduction of E6 and E7, which are two viral oncoproteins that are very relevant in transformation and carcinogenesis processes [99,102,103,104]. The decrease of STAT3 in cervical tumor cells has a drastic effect, inducing an increase in the expression of cell cycle control proteins such as p21, pRB, and p53, showing a decrease in cyclin D1 expression with an increase in the induction of apoptosis, which is produced by a decrease in pro-apoptotic proteins and an increase in the activation of effector caspases [99,104].

In summary, the inhibition of STAT3 in tumor cells results in a decrease in the E6 and E7 oncoproteins. The lack of these oncoproteins promotes an increase in pRB and p53, which are the proteins responsible for the inhibition and arrest of the cell cycle and the promotion of apoptosis. The inhibition of STAT3 highlights its importance as a therapeutic target in the treatment of cervical cancer. Thus, analysis of STAT3 and HPV E6/E7 oncoproteins is proposed as a new method for the detection of cervical lesions, in order to determine the severity of a lesion and to establish a survival prognosis [105].

#### 5.1.4. STAT4

STAT4 is expressed in the spleen, thymus, testes, and cells of the immune system, mainly in T cells. STAT4 is phosphorylated mainly by the action of IL-12, an antitumor cytokine [106,107]. Concerning the role of STAT4 in cancer, there are reports in ovarian cancer showing that STAT4 expression is associated with a poor prognosis and metastasis [108]. However, one report shows that STAT4 can favor tumor elimination; Anderson et al. showed that STAT4 plays an important role in the control of metastasis in head and neck squamous cell carcinoma (HNSCC). In their models, they showed that STAT4-deficient mice have a significant decrease in their Th1 and Th17 profiles and tumor cytotoxic activity. Additionally, STAT4 deficiency induces a higher rate of metastasis to lymph nodes and lungs, which led the authors to suggest that STAT4 mediates the resistance to metastasis of HNSCC and that the activation of STAT4 could potentially mitigate lymphatic metastasis [109]. Additionally, Nishi et al. analyzed gastric cancer samples and found that the expression of STAT4 mRNA correlates with the expression of IFNγ mRNA and that the disease-free survival at five years is higher in patients with a high expression of STAT4 compared with patients with a low expression (77.8% and 56.4%, respectively) [110]. Bioinformatic analysis in breast cancer has shown that high expression of IL-12/STAT4 axis molecules, such as the IL-12 receptor genes (*IL-12RB1* and *IL-12RB2*), STAT4, IFNγ, and TBX21, significantly increases the survival rates of patients with breast cancer, especially the more aggressive subtypes [111].

However, the role of STAT4 in cervical cancer has been poorly studied. Luo et al. measured the expression of STAT4 in histological lesions and found a higher expression of STAT4 in samples of adenocarcinoma and squamous cell carcinoma (64.71% and 66.54% respectively) compared with non-cancerous lesions such as chronic cervicitis and acuminate condyloma (25.93% and 28.57%, respectively). Moreover, they mentioned that 95.83% of the cancer tissue samples with metastases in the lymph nodes were positive for the expression of STAT4; they also mentioned an association between the expression of STAT4 with tumorigenesis and the severity of cervical lesions [112]. Interestingly, Sierra et al. found that the expression of genes such as *STAT1*, *STAT2*, *STAT3*, and *STAT4* increased in the skin of transgenic K14E7 mice, which implicates E7 in the overexpression of STAT4 [113]. Together, these data indicate a possible relationship between STAT4, progression, and metastasis in cervical tumor cells, as well as the role of oncoproteins such as E7 on the expression of STAT4. However, there are minimal preceding reports; therefore, more studies are needed to understand the role of STAT4 in cervical cancer.

The reason for the high expression of STAT4 in cervical cancer being associated with a poor response is something that should be investigated. However, it is important to note that STAT4 can form heterodimers with STAT1 and STAT3, and this could play a regulatory role in the immune response. For example, it is known that STAT4/STAT1 can form heterodimers in response to IL-35, an immunosuppressive cytokine associated with the generation of Tregs and with poor prognosis in some types of cancer. It is possible that the STAT4/STAT1 heterodimer may be related to anti-inflammatory programs [114,115,116,117]. There are intrinsic factors responsible for such vast plasticity to the transcriptional outcome generated by the JAK/STAT signaling pathway, and thus the response elicited by a cell or tissue. We can assume that antagonizing or synergistic effects can happen between STAT members, thus it should be considered that screening for the activity of one member of the STAT family might not be enough to predict disease progression. Possibly, we should analyze the balance between the interacting members of the STAT family.

#### 5.1.5. STAT5

STAT5 consists of two highly related proteins, STAT5a and STAT5b, with 90% homology. The STAT5a/b target genes are involved in proliferation, survival, differentiation, and apoptosis. STAT5 is necessary for the activation of immune system cells because it regulates the transcription of target genes such as the IL-2 α receptor (*IL-2RA*) (CD25), necessary for the formation of the high-affinity receptor (IL-2Rαβγ), and IL-2 signaling, essential for G1 phase progression and for mounting an effective immune response [118]. STAT5 is aberrantly active in various cancers, such as melanoma, glioblastoma multiforme, leukemia, and prostate and colorectal cancers, as well as in myeloid and lymphoproliferative diseases, among others. Activation of STAT5 is associated with a poor prognosis in most cases [119,120,121,122,123,124,125,126,127]. However, Chen et al. showed that the constitutive activation of STAT5 correlates with survival free of metastasis, as well as with overall survival in cervical cancer patients who received radiation therapy [128]. These data are consistent with some observations showing that STAT5 overexpression in lung cancer and breast cancer is associated with favorable overall survival after treatment [129,130,131,132].

In general, STAT5 increases significantly in most cervical tumors. However, there is a different pattern between the expression of the STAT5a and STAT5b isoforms. Sobti et al. revealed a higher expression pattern of STAT5b and an association with disease severity, while STAT5a was shown to be significantly downregulated in cervical tumor tissues. Furthermore, they found an association between HPV infection in cervical cancer cases and STAT5 overexpression [133]. Morgan and Macdonald found that STAT5 phosphorylation increases with the degree of cervical injury from CIN1 to CIN3. They observed an increase in STAT5 phosphorylation in the HPV16+ and HPV18+ cervical cancer cell lines compared with HPV-negative cervical cancer cells. Furthermore, they demonstrated that inhibition of STAT5b induces a significant reduction in cell proliferation due to a reduction in the expression of cyclin D1 and increased expression of p21. Additionally, they observed an increase in the induction of early and late apoptosis due to caspase activation, PARP cleavage, and a decrease in the expression of the antiapoptotic protein Bcl-xL. Interestingly, inhibition of STAT5b with specific STAT5b siRNA resulted in a reduction in the expression of the HPV E6 and E7 oncoproteins (the same pattern shown when STAT3 is inhibited using specific STAT3 siRNA, STAT3 inhibitor cryptotanshinone, or specific JAK2 siRNA). These observations imply an essential role of STAT5b in the oncogenic process and that its expression can regulate viral proteins [104,134]. Preceding studies show that the oncoprotein E7 can activate the phosphorylation of STAT5 and that this phosphorylation is necessary for the amplification of the viral genome of HPV31 [135]. In addition to the relationship between oncoproteins and STAT5, some reports show that cervical tumor cells are capable of generating strategies to positively regulate STAT5 activity. For example, there are reports that cervical tumor cells express the IL-2 and erythropoietin receptors and are capable of secreting IL-2 and erythropoietin to stimulate autocrine growth [136,137,138,139]. These results show that stimulation of cervical tumor cells with low doses of IL-2 or erythropoietin promotes the activation of STAT5 and cell proliferation. These data show the importance of STAT5 in the development of cervical cancer, as well as a link between STAT5 and viral oncoproteins. However, these stimuli can also promote the activation of other STATs such as STAT3.

The HPV E6 and E7 oncoproteins play an essential role in the activation of STAT3 and STAT5. Evidence shows that E6 induces the phosphorylation of JAK2-activating STAT3 and STAT5; an increase in the activation of both proteins correlates with the severity of a lesion, and their silencing affects the decrease in the viral oncoproteins E6 and E7. We know that STAT3 and STAT5 can form heterodimers and that they can bind different consensus sequences, and this affects gene expression; however, there are no reports that show the role of STAT3/STAT5 heterodimers and their contribution to cell transformation [100,104,134,140].

#### 5.1.6. STAT6

In T cells, STAT6 is essential for the development of Th2 cells; in B cells, it promotes the change of immunoglobulins into IgE and IgG1; meanwhile, in macrophages, it promotes M2 polarization induced by IL-4. STAT6 also works in other tissues, such as the mammary glands (promoting luminal mammary epithelial development), lungs (mediating the effect of IL-13 for mucus production), and skin (regulating epidermal differentiation genes). STAT6 is activated in response to IL-4 and IFN type 1; it functions as a homodimer or a heterodimer, and as both a negative and a positive regulator of transcription [141,142,143,144].

The role of STAT6 in cancer has recently been studied; there are studies in colorectal cancer, prostate cancer, glioblastoma, gastric cancer, and breast cancer that indicate that STAT6 may have an important role in the growth, aggressiveness, resistance to apoptosis, epithelial to mesenchymal transition, and metastasis of cancer cells [145,146,147,148,149]. However, some research groups suggest that STAT6 is necessary to inhibit growth and to mediate apoptosis via IL-4 in melanoma, colorectal, and breast cancer cells [150,151,152].

In cervical cancer, very few reports exist that indicate the role of STAT6. The group of Li showed that cervical tumor cells treated with IFN-γ/TNF-α co-immobilized in nanoparticles increased the expression and activation of STAT6. Moreover, they observed in their tumor models that the activation of STAT6 correlates with smaller tumor sizes and better survival. Furthermore, they showed that the silencing of STAT6 in HeLa cells reduces the induction of apoptosis and the expression of p53 and Bax. These data could indicate an essential role of STAT6 in the induction of cell death via IFN-γ and TNF-α [153]. Additionally, some reports show that viral proteins affect the expression of STAT6. The viral oncoproteins E6 and E7 are capable of promoting an increase in the expression of STAT6, as well as its activation. In one study, non-small cell lung cancer cells were transfected with vectors harboring HPV-16 E6 or E7 and it was observed that both E6 and E7 promoted an increase in the activation of STAT6 and STAT2, while for STAT5 and STAT3, they only increased their activation with E6 [154]. Other reports show that cells transfected with the HPV16 genome that contain a translation termination codon at amino acid 11 in the E5 open reading frame (E5 stop) increase STAT6 and STAT1 activation compared with human keratinocytes with the intact HPV16 genome. These data show that HPVE5 proteins modulate STAT6 and STAT1 activation [9]. It has also been observed that hepatoma cells are capable of forming STAT2/STAT6 heterodimers in response to IFN type I; moreover, B cells stimulated with IFNα induce the formation of a STAT2/STAT6/IRF9 complex. However, a decrease in STAT6 activation is associated with B cell resistance to the antiproliferative effects of IFNα [143,144]. However, there are very few studies in this regard, and more research must be generated to determine if the activation of the STAT6 homodimer or the STAT6/STAT2 heterodimers can contribute to the process of tumor recognition and elimination.

## 6. SOCS and Cervical Cancer

SOCS is a large family of proteins that negatively regulate JAK/STAT signaling. In cancer of the liver, lung, prostate, ovary, and breast, among others, there are antecedents that the reduction or silencing of one or more SOCS correlates with tumor progression and poor overall survival [155,156,157,158,159]. In cervical cancer, HPV can modulate the expression of different SOCS. For example, cervical cell lines positive for HPV16 and HPV18 show a decrease in the expression of SOCS1, SOCS3, and SOCS5 [160]. A decrease in the expression of SOCS1 has also been observed in precancerous lesions and in the different stages of cervical cancer; this decrease in the expression of SOCS1 apparently correlates with the severity of the lesion [161]. Different reports show that the inactivation of the *SOCS1* gene is due to the hypermethylation of its promoter, which the HPV virus can modulate [160,161]. It is known that SOCS1 can interact with the E7 protein and induces its ubiquitination and degradation, which, in turn, induces an increase in Rb protein and a decrease in tumor proliferation [162]. These data suggest that SOCS1 plays an important role in the regulation of E7 protein levels and its transformation potential. However, the role of SOCS1 remains controversial because the overexpression of SOCS1 in cervical tumor cells confers radioresistance [160].

## 7. Inhibition of the JAK/STAT Pathway as a Therapeutic Target in Cervical Cancer

STAT3 and STAT5 probably have the most critical roles in the development of cervical cancer; they are essential for proliferation and survival, in addition to being highly associated with tumor malignancy. The direct or indirect inhibition of JAK2, STAT3, and STAT5 has a high impact on the inhibition of proliferation, cell cycle arrest, and induction of apoptosis. There are a wide variety of inhibitors of the JAK/STAT pathway; however, STAT3 is of greater importance in most cancers, which is why it has a more significant number of inhibitors undergoing clinical trials. The STAT3 inhibitors found in clinical trials according to http://clinicaltrials.gov/ are AZD9150, Napabucasin, OPB-31121, WP1066, and TTI-101. For JAK proteins, they are TQ05105 (JAK2), Ruxolitinib (JAK1/2), BMS -911543 (JAK2), AZD1480 BID (JAK1/2), SB1518 (JAK2), Pacritinib (JAK2), XL019 (JAK2), SB1518 (JAK2), and CEP-701 (JAK2). However, these inhibitors are not specific to STAT3, and they also inhibit STAT5.

In cervical cancer, clinical trials are required to find out whether the use of these molecules can have a positive effect on the overall and disease-free survival of patients, in addition to evaluating the toxicity of these treatments, as the reports indicate that STAT3 inhibitors are highly toxic. Experimental data in cervical tumor cells show that JAK2, STAT3, and STAT5 inhibitors reduce cell proliferation, induce apoptosis, and improve response to other drugs such as cisplatin [99,163,164,165,166]. Strategies to inhibit or reduce STAT3 activation include the use of interfering STAT3 siRNAs, which reduce the resistance of cervical tumor cells to cisplatin treatment. Additionally, the use of some compounds such as propofol, arctigenin, and mahanin improves the induction of cell death by inhibiting STAT3 [163,164,165,166,167,168]. The JAK/STAT pathway participates in immune activation and regulation processes, including those involved in tumor cell recognition and tumor-driven immune escape. In this context, natural killer (NK) cells are an essential component of the innate immune system, capable of providing a rapid and efficient response against virus-infected and tumor cells. The response to different cytokines and the activation of the JAK/STAT pathway are important in various processes; for example, STAT1, STAT4, and STAT5 stimulate the maturation and cytotoxicity of NK cells, while STAT3 and STAT6 seem to have a suppressive role in NK cell activity [169]. These different responses could explain why the inhibition of the JAK/STAT pathway may have unexpected effects, such as those shown by Bottos et al., who found that the inhibition of JAKs with NVP-BSK805 and ruxolitinib (JAK1/2 inhibitor) in experimental models of breast cancer improved metastasis by inhibiting the maturation, activation, proliferation, and cytotoxicity of NK cells [170]. The inhibitor ruxolitinib reduces Treg cell levels and decreases dendritic cell differentiation, migration, and activation, in addition to increasing the rate of infections [171,172,173]. These findings stress the importance of analyzing the adverse effects of the inhibitors of the JAK/STAT pathway on cancer immunosurveillance. Thus, it is necessary to find targets that affect tumor cells and facilitate the activation of the immune system.

## 8. Concluding Remarks

The JAK/STAT pathway mediates a plethora of regulatory processes of the immune system, including many processes that are involved in tumor cell recognition and tumor-driven immune escape. Therefore, understanding the function, redundancy, and connectedness of the components of the JAK/STAT pathway in cancer is essential in the fight against solid tumor growth and metastases. As a consequence of the activation of JAK kinases, STAT hyperactivation is present in different types of cancer, such as cervical cancer, which remains a public health problem in developing countries. Most cervical carcinomas are related to HPV infection; in particular, the presence of the oncoproteins E6 and E7 is necessary for the establishment and progression starting from premalignant lesions. As shown above, these two oncoproteins are involved in the dysregulation of the JAK/STAT pathway; thus, it is not surprising that this signaling pathway has become an attractive therapeutic target in modern medicine. However, many challenges remain in elucidating how this conserved pathway and its pleiotropic nature regulate the proliferation and survival of cervical cancer cells.

## Figures and Tables

**Table 1:** Cytokine receptors and janus kinases (JAKs).

Type I Receptors	Shared γc subunit	IL-2R, IL-4R, IL-7R, IL-9R, IL-15R, and IL-21R	JAK1, JAK2, and JAK3
	Shared gp130 subunit	IL6R, IL-11R, OSMR, LIFR, CNTFR, and IL-27R	JAK1, JAK2, and TYK2
	Shared βc subunit	IL-3R, IL-5R, and GM-CSFR	JAK2
	Shared IL-12Rβ1 subunit	IL-12R and IL-23R	TYK2 and JAK2
	Homodimeric cytokine receptors	EPOR, G-CSFR, GHR, PRLR, TPOR, and LEPR	JAK1 and JAK2
Type II Receptors	Interferon receptors	IFNα, IFNβ, IFNγ, IL-10R, IL-19R, IL-20R, IL-22R, IL-24R, IL-28R, and IL-29R	JAK1, JAK2, and TYK2
